# Common Mental Disorder and Its Associated Factors among Prisoners in North Wollo Zone Correctional Institutions, Northeastern Ethiopia

**DOI:** 10.1155/2022/8980774

**Published:** 2022-12-16

**Authors:** Kindie Mekuria Tegegne, Teshome Gebremeskel Aragie, Mekonnin Tesfa Lemma, Yossef Teshome Zikarg, Solomon Moges Demeke

**Affiliations:** ^1^Woldia University, College of Health Science, Department of Psychiatry, P.O Box 400, Woldia, Ethiopia; ^2^Woldia University, College of Health Science, Department of Anatomy, P.O Box 400, Woldia, Ethiopia; ^3^Woldia University, College of Health Science, Department of Physiology, P.O Box 400, Woldia, Ethiopia; ^4^Addis Ababa University, College of Health Science, School of Medicine, Department of Anatomy, P.O Box 9086, Addis Ababa, Ethiopia

## Abstract

**Background:**

Globally, about 450 million people suffer from mental disorders of which about 11% are assumed to be prisoners. The presence of mental illness among prisoners contributes to an increase in the risk of suicide, violence, morbidity, and mortality. In Ethiopia, there is a paucity of data particularly from resource-limited areas to assist policy maker's efforts in reforming mental health care.

**Objective:**

This study is aimed at assessing common mental disorders and its associated factors among prisoners in North Wollo zone correctional institutions, Northeastern Ethiopia.

**Method:**

Institution-based cross-sectional study was conducted on 401 study participants from January–February 2021. A simple random sampling technique was employed to enroll the study participants. Structured interviewer-administered Self Reporting Questionnaires-20 (SRQ-20) developed by the World Health Organization was used to collect the data. Data were checked for completeness, coded and entered into Epi data version 4.2, and transferred to SPSS version 23.0 for final analysis. Binary logistic regression analysis was carried out to identify factors associated with common mental disorders. Statistical significance was declared at *p* values < 0.05 in the final model.

**Results:**

The prevalence of common mental disorders was found to be 63.6% (95% CI 58.9, 68.3). After adjusting for confounding factors using multiple logistic regression, having children (AOR = 3.7, 95% CI: 1.93, 7.36), poor social support (AOR = 6.6, 95% CI: 2.93, 14.93), history of mental illness (AOR = 6.5, 95% CI: 1.78, 24.3), one- to five-year prison stay (AOR = 2.6, 95% CI: 1.38, 5.04), greater than five-year prison stay (AOR = 5.7, 95% CI: 2.05, 16.27), experiencing one stressful life event (AOR = 5.2, 95% CI: 1.83, 15.01), experiencing two or more stressful life events (AOR = 7.3, 95% CI: 2.98, 17.9), unavailability of reading materials (AOR = 4.3, 95% CI: 1.63, 11.43), and greater than or equal to eleven years of sentences (AOR = 4.4, 95 CI: 1.82, 10.70) were factors significantly associated with common mental disorders.

**Conclusion:**

Common mental disorders are highly prevalent among prisoners in this study area. The result of this study suggests the importance of screening and providing psychiatric counseling to this highly vulnerable population.

## 1. Introduction

Common mental disorder (CMD) is a group of noncommunicable diseases [1]. Common mental disorder is a term used to describe diseases including depression, anxiety, and somatoform disorder, which is characterized by symptoms such as insomnia, fatigue, irritability, forgetfulness, difficulty in concentrating, and somatic complaints [2]. These disorders are the most prevalent mental disorders in the world. Although they are not as severe as psychotic disorders, they can pose a significant public health problem because of their high prevalence and serious effects on personal wellbeing, family, work, and use of health services [3, 4].

Mental disorders are nowadays becoming a global public health agenda. Worldwide, significant populations are suffering from CMDs [5]. Approximately 450 million people suffer from mental disorders worldwide [6]. Thus, it is becoming the second leading cause of health disability worldwide. Studies revealed that the overall burden of common mental disorders among prisoners ranges from 13 to 92.5% worldwide [7, 8]. Prison is a correctional institution in which prisoners live under limited liberty, autonomy, and communication with family and friends. It is a place where these mental disorders are becoming a significant health problem [9]. Although the causes of most mental disorders are not fully understood concerns about separation from family or friends, violence, lack of privacy, isolation from social networks, insecurity about future prospects, term of sentence, inadequate health services, and lack of social support are identified as some of the factors that contributed to the development of mental health problems in prison [6, 10]. Different studies found that risk factors for CMDs vary across different settings; thus, findings in one area may not apply to other areas [11–14]. Common mental disorders significantly impaired the patients' quality of life, worsening their physical symptoms, and more likely to face ongoing stress [15]. It is suggested that the presence of mental illness among prisoners contributed to an increased risk of suicide, violence, morbidity, and mortality [16, 17]. It is believed that the burden of common mental disorders is worst in low- and middle-income countries where the impact of poverty and other communicable diseases are highly rampant. Out of people suffering from depression, 85% live in low- and middle-income countries [18]. Attention is not given to timely mental disorders screening and treatment in many prisons mainly in developing countries [19]. There is also a paucity of data regarding risk factors of mental disorders among prisoners in low-income countries, including Ethiopia, despite the existence of high numbers of prisoners. Thus, this study is aimed at determining the prevalence of common mental disorders and its associated factors among North Wollo zone correctional institutions, Northeastern Ethiopia.

## 2. Method and Materials

### 2.1. Study Area and Period

The study was conducted in North Wollo zone correctional institutions from January–February 2021. North Wollo zone is one of the 10 zones of the Amhara Region of northern Ethiopia. It is bordered on the south by South Wollo, on the west by South Gondar, on the north by Waghemra and Tigray Region, and on the east by Afar Region. According to the data obtained from the prison administrator, North Wollo zone has two correctional institutions, which are located in Woldia and Lalibela towns, with a total of 1326 prisoners at the time of data collection.

### 2.2. Study Design and Study Population

An institution-based cross-sectional study design was employed. The study population was prisoners in the North Wollo zone correctional institutions. Randomly selected prisoners were the study units. Prisoners sentenced for their proven guilty were included in this study, whereas prisoners with known mental illness and unable to communicate were excluded.

#### 2.2.1. Inclusion Criteria and Exclusion Criteria

*(1) Inclusion*. All prisoners who were imprisoned at North Wollo Zonal correctional institutions.

*(2) Exclusion*. Prisoners who were critically ill and on follow-up associated with psychiatric disorders were excluded from the study.

### 2.3. Sample Size Determination and Sampling Technique

The sample size was determined using a single population proportion formula by considering the assumption of *Zα*/2 = critical value for normal distribution at 95% confidence level, which equals to 1.96 (*z* value at *α* = 0.05), *P* (estimated proportion) = 58.4%, which was taken from the previous study conducted in Addis Ababa [20] and margin of error 5% (*d* = 0.05). The following formula was used to calculate sample size:

(1) $$\begin{array}{c} n=\frac{{\left(Za/2\right)}^{2}*p\left(1-p\right)}{d^{2}}, \end{array}$$

(2) $$\begin{array}{c} n=\frac{{\left(1.96\right)}^{2}*0.58\left(0.42\right)}{{\left(0.05\right)}^{2}}=375. \end{array}$$

Adding 10% nonresponse rate, the final sample size was = 413.

**Figure 1:**
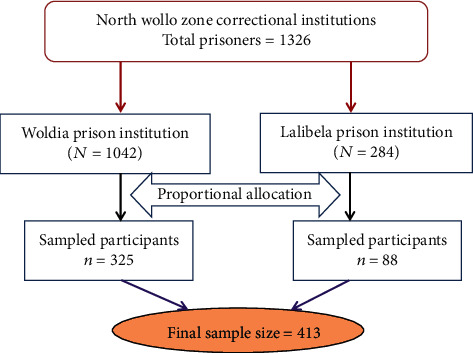
Depicts the sampling procedure of North Wollo zone correctional institutions, Northeastern Ethiopia.

A complete list of prisoners was obtained from the zonal prison administration office. To reduce the chance of selection bias, simple random sampling technique was used to select the study participants. The sample was proportionally allocated to each prison institution (Figure 1). The sampling procedure is presented as follows.

### 2.4. Data Collection Instrument, Technique, and Data Quality Control

The study used an interviewer-administered structured questionnaire to collect data about sociodemographic characteristics, substance use, clinical, and prisoner-related characteristics. Data were collected through face-to-face interview. Common mental disorders were assessed using Self Reporting Questionnaires-20 (SRQ-20), developed by the World Health Organization to screen common mental disorders [21]. Oslo social support scale is a tool that assess about the social relationship of an interviewee, and it is Likert scale, ranging from 3–14 points [22]. To ensure the reliability of the tool, Cronbach's alpha was used. The psychometric properties of both SRQ-20 and Oslo-social support scale were checked its reliability using Cronbach's alpha. The findings indicated that SRQ-20 had a coefficient of 0.92, and Oslo-social support had a coefficient of 0.81. Both tools we have used depicted that the value of Cronbach's alpha is above the suggested value of 0.7; thus, the study was reliable [23]. Based on the reliability test, it was concluded that the scale used in this study is reliable to capture the constructs as shown in Table 1.

Four BSc Nurses were involved in the data collection, and it was supervised by two masters of public health professional. The training was given to data collectors before the actual data collection regarding the purpose of the study, data collection procedures, and ethical issues during data collection. The questionnaire was initially developed in English then translated into working language (Amharic) and translated back into English language for keeping its consistency. Before the actual data collection, the tool was pretested on 5% of the total sample size in Hayik correctional institution. The completeness of the data and consistency was checked daily throughout the data collection period.

### 2.5. Operational Definition

1. *Common Mental Disorders*. Refers to the most prevalent conditions classified under depressive episodes, anxiety, and somatic symptoms. A score of eight or more in SRQ-20 in the past 4 weeks will be considered as having common mental disorders [24]
2. *Chronic Medical Illness*. At least one of these chronic diseases: HIV/AIDS, diabetes, cancer, asthma, hypertension, and heart disease [25]
3. *Social Support*. Assessed by the Oslo 3-item social support scale. The sum score scale ranges from 3 to 14 with three broad categories: “poor support” 3–8, “moderate support” 9–11, and “strong support” 12–14 [26]
4. *Presence of Stressful Life Events*. Individuals who had at least one or more stressful life events (close family member died, divorce, serious illness or injury in the family member, etc.) in the last four weeks [27]

### 2.6. Data Processing and Analysis

Data were checked for completeness, coded, and entered into EPI-data version 4.2.0 and exported to SPSS version 23.0 for final analysis. Descriptive statistics were carried out to compute frequencies, percentages, and proportions of sociodemographic characteristics. To measure the possible association of factors with the outcome variable, bivariable and multivariable logistic regression was computed. Bivariable analysis was performed to select candidate variables at *p* value less than 0.25. Variables with *p* value of less than 0.25 were transferred to multivariable logistic regression model to identify factors independently associated with common mental disorders. Variables with *p* value of less than 0.05 in multivariable logistic regression were considered statistically significant. Adjusted odds ratios with 95% CI were used to show the strength of association between outcome and predictor variables.

## 3. Results

### 3.1. Sociodemographic Characteristics of Respondents

Out of 413 participants, 401 completely responded to the questionnaire making the response rate of 97%. Among these, most of the respondents 386 (96.3) were males. About 336 (83.8%) were literates. The majority of the participants 386 (96.3%) belonged to Amhara ethnic group. Most of the prisoners 346 (86.3%) were Orthodox Christian followed by Muslims 46 (11.1%) (Table 2).

### 3.2. Psychosocial and Other Characteristics of Prisoners

In this study, more than half 223 (55.6%) of the study participants had poor social support. Nearly three fourth of the respondents 289 (72.1%) have experienced two or more stressful life events while 64 (16%) faced only one stressful life event. The majority of the prisoners 299 (89%) have the desire to use reading materials to cope up their stressful life events. However, 265 (88.6%) of the prisoners responded that there is a shortage of reading materials to read in the prison institution (Table 3).

### 3.3. Prevalence of Common Mental Disorders and Its Associated Factors

**Figure 2:**
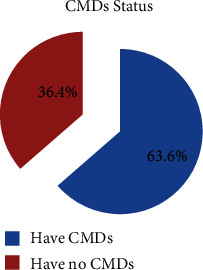
Prevalence of common mental disorders among prisoners in North Wollo zone correctional institutions, Northeastern Ethiopia.

**Figure 3:**
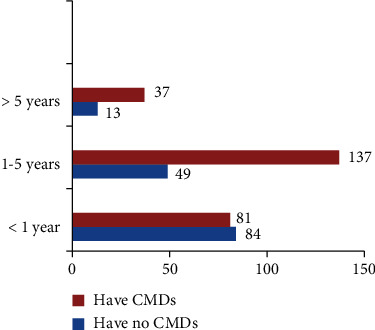
Duration of prison stay among prisoners in North Wollo zone correctional institutions, Northeastern Ethiopia.

The prevalence of common mental disorders among prisoners in North Wollo zone correctional institutions using SRQ-20 scale with cutoff point 8 and above was found to be 63.6% (95% CI 58.9, 68.3) (Figure 2). Almost one-third of the respondents, 137 (34.1%) were incarcerated for one to five years and had common mental disorders (Figure 3). In the bivariable logistic regression analysis, age, residence, illiteracy, duration of a prison stay, social support, stressful life events, current medical illness, types of crime, occupation, chat chewing, cigarette smoking, history of mental illness, unavailability of sufficient reading materials, and duration of a sentence were associated with the common mental disorders. Those variables that have a *p* value less than or equal to 0.25 were entered into a multivariable logistic regression model to adjust for possible confounders.

In multivariate logistic regression analysis, having children, history of mental illness, longer duration of a prison stay, experiencing stressful life events, poor social support, duration of sentence, and unavailability of reading materials were significantly associated with the common mental disorders. Accordingly, prisoners having children were 3.7 (AOR = 3.7, 95% CI: 1.93, 7.36) times more likely to develop common mental disorders. The odds of developing common mental disorders among prisoners having poor social support were 6.6 (95% CI: 2.93, 14.93) higher when compared to respondents with good social support. Study participants who stayed 1-5 years and greater than 5 years in prison were 2.6 and 5.7 times more likely to develop common mental disorders than prisoners who stayed less than 1 year in prison, respectively. Duration of sentence was also a significant predictor of CMDs. The odds of developing common mental disorders among respondents who have a history of mental illness were 6.5 (95% CI: 1.78, 24.3) higher when compared to individuals who have no history of mental illness. Prisoners experiencing two or more stressful life events were 7.3 (95% CI: 2.98, 17.9) times more likely to develop common mental disorders compared to prisoners who have no stressful live events (Table 4).

## 4. Discussion

The present study was aimed at assessing the prevalence of common mental disorders and its association with various risk factors. Thus, the prevalence of common mental disorders (CMDs), in our study, was found to be 63.6% (95% CI: 58.9, 68.3). This finding is similar with the studies conducted in Jimma correctional institution 62.70% [14], Debre-Markos 67.6% [28], Zambia 63.10% [29], Kenya 63.2% [12], and England 65.3% [9].

Our prevalence is significantly higher when compared to studies conducted in Kombolcha 32.4% [27], Addis Ababa 58.4% [20], Nigeria 57% [30], South Africa 55.4% [16], Egypt 22% [10], and Iran 43.4% [31]. The disparity could be due to differences in socioeconomic status, study setting, sample size, prison environment, and prisoner handling. For example, the Kombolcha study was a community-based survey, indicating a lower prevalence of CMDs. CMDs are more common among prisoners than in the general population, according to studies in low-income countries. One possible explanation is that prisoners live in a stressful environment, are separated from their families, and have limited freedom of movement, which increases the risk of developing CMDs [12, 32–34].

On the other hand, the finding of our study is lower than those of studies done in South Wales [33], Australia [35], and Pakistan [36] where the magnitude was found to be 74%, 80%, and 85%, respectively. The discrepancy might be attributed to a difference in measurement tools and sample sizes. For instance, studies were done in Australia and South Wales included study participants that are more than twice as many as our study participants, and they also used the composite international diagnostic interview (CIDI) to assess mental illnesses.

Regarding the types of crime, the prisoner who committed rape was less likely to develop common mental disorders compared to prisoners who committed theft and murder. The reason might be due to the conditions, which should be correlated in the local context of the societal cultural perspective regarding to rape compared to other crimes like murder and theft. Even though, the authors did not have concrete justification regarding to the societal perception on rape, most of the societies living there believe that sexual assault, harassment, and rape are not that much considered as huge crime for them compared to other crimes like murder. Additionally, the authors merged sexual assault, sexual harassment, and rape as one variable rape on regression. Thus, most of the prisoners who committed sexual assault and harassment may consider their action as less than other crimes like murder and theft.

Mental disorders are regarded as complex, and its causes vary depending on the particular disorder and the individual. Although the causes of most mental disorders are not fully understood, researchers have identified a variety of biological, psychological, and environmental factors that can contribute to the development or progression of mental disorders [6, 19].

Our study has also tried to identify the factors for common mental disorders among prisoners. Accordingly, having mental disorders was significantly associated with poor social support. Thus, prisoners with poor social support were 6.6 times more likely to develop CMD when compared to prisoners with good social support. The reason might be social support would improve the wellbeing of those under stress by acting as a buffer or moderator of that stress. It is also believed that perceived social support can enhance help-seeking and coping mechanisms through positive appraisal of the situation and reduction in negative emotional responses [37]. Our finding is consistent with studies done in Jimma [14], Kombolcha [27], Addis Ababa [20], Egypt [10], and Iran [31].

Our study also found that the odds of suffering CMDs are higher among prisoners experiencing one or more stressful life events. This is in line with two studies done in Ethiopia [11, 27]. Stressful life events have a substantial causal relationship with the onset of CMDs [38]. Study also showed that CMDs were associated with stressful life events related to family, work, social isolation, chronic physical illness, and lifestyle pressures [27]. The possible explanation is that the acquired abnormalities in the stress response and stress-induced structural change in brain regions may induce due to changes in dendritic and synaptic structure within brain part [39]. Moreover, the higher the number of stressful life events that the person encounters, the higher the chance of CMD development [38].

The current finding revealed that a longer duration of prison stay was associated with CMDs. The odds of developing CMDs among prisoners who stayed in the prison for more than five years were 5.7 higher than those who stayed for less than one year. Similarly, the duration of a sentence was also a significant predictor of CMDs. This finding is supported by a study done in Debre Berhan [11] and Dilla [13]. Researchers suggest that when the prisoners stay in prison longer, they feel as they are detached from the external social role and physical world and perceive themselves as born to be imprisoned. As they stay more in the prison, they will be exposed to a prison environment that is crowded and shabby physical setting, despotic rules, and regulations, and they broke and closed interpersonal relationships [19, 35, 38]. These would possibly escalate the vulnerability to experience mental disorders.

The unavailability of sufficient reading materials in the prison was another factor significantly associated with CMDs. Thus, prisoners who have no reading materials were more prone to develop common mental disorders when compared to their counterparts. Providing emphasis and new understanding of interest to read and see libraries at the correctional institution creates important impact of prisoner health [40]. For instance, our assessment shows that about 89% of literate prisoners have a desire to read materials to relax and cope with stressful life events. It is believed that during reading, people shift their focus and forget about stressful life events they encounter. As a result, prisoners' chance to develop CMDs decreases.

Our result also suggests that having children was an independent predictor of CMDs. Prisoners who had children were nearly four times higher to suffer CMDs when compared to their counterparts. This is probably because prisoners who had children might be concerned about their children; thus, higher risk to experience mental illness. In contrast, a couple of studies conducted in Southwestern parts of Ethiopia Jimma [14, 41] did not find an association between having children and CMDs. The reason may be related to a difference in sample size. Having a history of mental illness was also another factor independently associated with CMDs. This is in line with studies done in Addis Ababa [20], Debre Markos [28], and Egypt [10]. This is possibly due to imprisonment might be added stress to prisoners' life that exacerbates their mental illness. The limitation of this study is that the obtained data were based on the subjective report by study participants, and this may lead to over or underestimation of the prevalence of CMDs.

## 5. Limitation of the Study

We used a cross-sectional study design in which it is difficult to establish cause and effects relationships. The authors fear of the presence of social desirability bias due to the presence of questions/tools, which can make the subjects systematically more likely to provide a socially acceptable response. For instance, tools regarding “substance use” are more likely affected by social desirability bias.

Recall bias could also be the main limitation of this study, as most of the tools which explore the individual's previous activity are liable to recall bias. Participants might be faced particular outcome or exposure may remember events more clearly or amplify their recollections than the others. Therefore, individuals who have more than one problem might magnify the one which challenge him/her and may ignore the remaining problems which might have effect on developing depression. For instance, tools regrading on “stressful life events” is more liable on recall bias.

## 6. Conclusions and Recommendations

The present study revealed that nearly two-thirds of prisoners in the North Wollo zone correctional institutions were suffering from common mental disorders. Having children, history of mental illness, longer duration of prison stay, experiencing one or more stressful life events, poor social support, duration of the sentence, and unavailability of sufficient reading materials were significantly associated with the common mental disorders. Therefore, the prison administrations need to strengthen mental health services in prisons. The prison institutions should facilitate in-service training for prison health professionals on early screening and prevention of adverse effects of mental disorders. Counselors and mental health experts should provide psychosocial support especially for prisoners who have poor social support, experienced stressful life events, and are sentenced for a long time.

## Figures and Tables

**Table 1:** Reliability test on SRQ-20(0.92) and Oslo-social support scale (0.81).

Reliability statistics		
Cronbach's alpha	Cronbach's alpha based on standardized items	*N* of items
0.920	0.919	20
0.818	0.817	12

**Table 2:** Sociodemographic characteristics of respondents in North Wollo zone correctional institutions, Northeast Ethiopia.

Characteristics	Category	Frequency	Percent
Age in years	18-27	173	43.1

	28-37	141	35.2
	38-47	44	11
	48-57	16	4
	≥58	27	6.7

Sex	Male	386	96.3
	Female	15	3.7

Marital status	Single	208	51.9
	Married	163	40.6
	Divorced	18	4.5
	Widowed	12	3.0

Residence	Urban	197	49.1
	Rural	204	50.9

Educational status	Cannot read and write	65	16.2
	Can read and write	336	83.8

Ethnicity	Amhara	386	96.3
	Oromo	8	2
	Othersꜛ	7	1.7

Religion	Orthodox	346	86.3
	Muslim	46	11.5
	Others^**⸸**^	9	2.2

ꜛTigre and Afar, ^⸸^protestant and catholic.

**Table 3:** Distribution of psychosocial and other characteristics of prisoners in North Wollo zone correctional institutions, Northeastern Ethiopia.

Characteristics	Category	Frequency	Percent
Social support	Poor	223	55.6
	Intermediate	113	28.1
	Good	65	16.2

Stressful life events	No SLE	48	12
	One SLE	64	16
	≥2 SLE	289	72.1

History of mental illness	Yes	53	13.2
	No	348	86.8

Family history of mental illness	Yes	35	8.7
	No	366	91.3

History of chronic illness	Yes	33	8.2
	No	368	91.8

History of chat chewing	Yes	129	32.2
	No	272	67.8

History of drinking alcohol	Yes	289	72.1
	No	112	27.9

History of smoking	Yes	90	22.4
	No	311	77.6

Duration of prison stay	<1 year	165	40.5
	1-5 years	186	46.3
	>5 years	50	12.4

Duration of sentences	≤5 years	172	42.8
	6-10 years	97	24.1
	≥11 years	132	32.8

Unavailability of reading materials	No	34	11.4
	Yes	265	88.6

Reading desire to cope up with stressful life events	No	37	11
	Yes	299	89

SLE: stressful life event.

**Table 4:** Bivariable and multivariable regression results on common mental disorder and its associated factors among prisoners in North Wollo zone prison institutions, Northeastern Ethiopia.

Variables		CMD (*n* = 401)		Odd ratios	
		No	Yes		
		146 (36.4)	255 (63.6)	COR (95% CI)	AOR (95% CI)
Age	18-27	66 (16.4%)	107 (26.6%)	1	1
	28-37	38 (9.4%)	103 (25.6%)	1.67 (1.03, 2.70)	1.12 (0.55, 2.29)
	38-47	18 (4.4%)	26 (6.48%)	0.89 (0.454, 1.74)	0.28 (0.106, 1.264**)**
	48-57	9 (2.24%)	7 (1.7%)	0.48 (0.17, 1.35)	0.063 (0.112, 1.323)
	≥58	15 (3.7%)	12 (2.9%)	0.49 (0.21, 1.11)	0.103 (0.026, 1.401)

Residence	Rural	61 (15.3%)	143 (35.6%)	1.7 (1.17, 2.68)	1.9 (0.4, 3.50)
	Urban	85 (21.1%)	112 (27.9%)	1	1

Having children	No	93 (23.1%)	104 (25.9%)	1	1
	Yes	53 (13.2%)	151 (37.6%)	0.39 (0.258, 0.597)	3.7 (1.93, 7.36)^∗^

Educational level	Illiterate	31 (7.7%)	34 (8.4%)	0.57 (0.33, 0.97)	0.40 (0.17, 1.92)
	Literate	115 (28.6%)	221 (55.1%)	1	1

Occupation	Gov't employee	17 (4.2)	33 (8.2)	1	1
	Farmer	53 (13.2)	104 (25.9)	1.01 (0.516, 1.98)	1.05 (0.348, 3.22)
	Merchant	40 (9.9)	56 (14.7)	0.72 (0.354, 1.47)	1.47 (0.642, 3.36)
	Unemployed	36 (8.9)	62 (15.4)	0.887 (0.43, 1.81)	1.02 (0.405, 2.57)

Chat chewing	No	112 (27.9)	160 (39.9)	1	1
	Yes	34 (8.4)	95 (23.6)	1.95 (1.23, 3.09)	0.94 (0.38, 2.36)

Drink alcohol	No	50 (12.4)	62 (15.4)	1	
	Yes	96 (23.9)	193 (48.1)	0.61 (0.395, 963)	0.98 (0.508, 1.90)

Types of crime	Theft	47 (11.7)	80 (19.95)	1.19 (0.66, 2.15)	1.65 (0.68, 4.00)
	Murder	57 (14.2)	119 (29.6)	1.47 (0.84, 2.56)	0.56 (0.225, 1.40)
	Rape	11 (2.7)	12 (2.9)	0.76 (0.30, 1.96)	0.20 (0.049, 0.88)^∗^
	Others	31 (7.7)	44 (10.9)	1	1

Duration of prison stay	<1 year	84 (20.4)	81 (20.1)	1	1
	1-5 years	49 (12.2)	137 (34.1)	2.8 (1.85, 4.53)	2.6 (1.38, 5.04)^∗^
	>5 years	13 (3.2)	37 (9.2)	2.9 (1.46, 5.95)	5.7 (2.05, 16.27)^∗^

Social support	Poor	53 (13.2)	170 (42.3)	5.13 (2.85, 9.23)	6.6 (2.93, 14.93)^∗^
	Intermediate	53 (13.2)	60 (14.9)	1.18 (0.97, 3.37)	2.2 (0.93, 5.28)
	Good	40 (9.9)	25 (6.2)	1	1

Duration of sentences	≤5 years	79 (19.7)	93 (23.1)	1	1
	6-10 years	32 (7.9)	65 (16.2)	1.7 (1.02, 2.89)	3.2 (1.40, 7.60)^∗^
	≥ 11 years	35 (8.7)	97 (24.1)	2.3 (1.44, 3.84)	4.4 (1.82, 10.70)^∗^

Stressful life event	No SLE	29 (7.2)	19 (4.7)	1	1
	One SLE	24 (5.9)	40 (9.9)	2.5 (1.17, 5.48)	5.2 (1.83, 15.01)^∗^
	≥2 SLE	93 (23.1)	196 (48.8)	3.2 (1.71, 6.03)	7.3 (2.98, 17.9)^∗^

History of mental illness	No	141 (35.1)	207 (51.6)	1	1
	Yes	5 (1.2)	48 (11.97)	6.5 (2.54, 16.8)	6.5 (1.78, 24.3)^∗^

Chronic medical illness	No	141 (35.1)	227 (56.6)	1	1
	Yes	5 (1.2)	28 (6.9)	3.47 (1.31, 9.21)	2.32 (0.87, 4.70)

Unavailability of reading materials	No	18 (6.0)	16 (5.2)	2.01 (0.98, 4.13)	4.3 (1.63, 11.43)^∗^
	Yes	95 (32.0)	170 (56.8)	1	1

SLE: stressful life event, ^∗^significant at *p* < 0.05. Others: corruption, human trafficking, and punching.

## Data Availability

The datasets analysed during the present study are not publicly available due to participants' private policies but are available from the corresponding author on reasonable request.
